# Understanding the Effect of Side Reactions on the Recyclability of Furan–Maleimide Resins Based on Thermoreversible Diels–Alder Network

**DOI:** 10.3390/polym15051106

**Published:** 2023-02-23

**Authors:** Brandon T. McReynolds, Kavon D. Mojtabai, Nicole Penners, Gaeun Kim, Samantha Lindholm, Youngmin Lee, John D. McCoy, Sanchari Chowdhury

**Affiliations:** 1 Department of Materials and Metallurgical Engineering, New Mexico Institute of Mining and Technology, Socorro, NM 87801, USA; 2 Department of Chemical Engineering, New Mexico Institute of Mining and Technology, Socorro, NM 87801, USA

**Keywords:** Diels–Alder reaction, furan, maleimide, recyclability, reversible, maleimide homopolymerization

## Abstract

We studied the effect of side reactions on the reversibility of epoxy with thermoreversible Diels–Alder (DA) cycloadducts based on furan and maleimide chemistry. The most common side reaction is the maleimide homopolymerization which introduces irreversible crosslinking in the network adversely affecting the recyclability. The main challenge is that the temperatures at which maleimide homopolymerization can occur are approximately the same as the temperatures at which retro-DA (rDA) reactions depolymerize the networks. Here we conducted detailed studies on three different strategies to minimize the effect of the side reaction. First, we controlled the ratio of maleimide to furan to reduce the concentration of maleimide groups which diminishes the effects of the side reaction. Second, we applied a radical-reaction inhibitor. Inclusion of hydroquinone, a known free radical scavenger, is found to retard the onset of the side reaction both in the temperature sweep and isothermal measurements. Finally, we employed a new trismaleimide precursor that has a lower maleimide concentration and reduces the rate of the side reaction. Our results provide insights into how to minimize formation of irreversible crosslinking by side reactions in reversible DA materials using maleimides, which is important for their application as novel self-healing, recyclable, and 3D-printable materials.

## 1. Introduction

Epoxies are an important class of thermoset polymer with high thermal stability, chemical resistance, and mechanical strength; applications of epoxies include structural materials, electronics, paints, and adhesives [1]. However, removal and recycling of conventional epoxy resin is challenging due to its irreversibly crosslinked nature. Irreversibly crosslinked epoxy polymers cannot be reshaped, reprocessed, or recycled due to their insoluble and non-meltable features. Implementation of thermally reversible Diels–Alder (DA) crosslinked network in epoxy is one of the popular choices for the development of self-healing and recyclable epoxies [2,3,4,5]. At higher temperatures (>120 °C), the DA bonds break as a result of the retro Diels–Alder reaction (rDA), which breaks down the 3D network structure into a flowable polymer melt. The forward Diels–Alder (fDA) reaction occurs at lower temperatures (<80 °C), which reforms the bonds and solidifies the epoxy again. Diels–Alder (DA) reactions between furan and maleimide have been widely applied in developing thermally recyclable epoxy due to the processing advantages of mild reaction conditions, few by-products, and catalyst-free requirements [6].

However, development of a truly recyclable epoxy employing the DA chemistry is challenging due to the irreversible crosslinking imparted by side reactions occurring at high temperatures (>110 °C). One of the most important and well-known side reactions is homopolymerization of maleimide moieties [7,8,9,10]. This has been evidenced from electron-spin resonance (ESR) studies to occur without initiator due to free radical generation of the maleimide itself, either through thermal homolysis of the C=C double bond or more likely a donor-acceptor complex of maleimides [11,12]. Another possible side reaction can occur between maleimide and amine groups left in the system through a Michael’s addition reaction [13]. In fact, many of the early works with (bis)maleimide resins studied their reactions with aliphatic amines, which were often used as chain extenders to reduce the brittleness of the thermosets prepared with the neat (bis)maleimides [10,14]. The recent history and future directions of bismaleimide research are well covered in a review [15]. In the context of DA materials, a popular choice for adding furan moieties to a prepolymer backbone is through the epoxy-amine reaction. This is typically a high conversion (>80%) reaction but can still be incomplete, leaving free amine in the system that could eventually react with the maleimide groups [13,16,17]. Although our focus is the maleimide homopolymerization, we expect that other irreversible crosslinks would impact the recyclability likewise. This puts an emphasis on checking for unreacted reactant and including purification steps in developing any reaction scheme. While the effect of these side reactions is often acknowledged as a limitation on the development of an epoxy capable of repeated recyclability, the detailed studies of these side reactions are very limited.

**Scheme 1:**
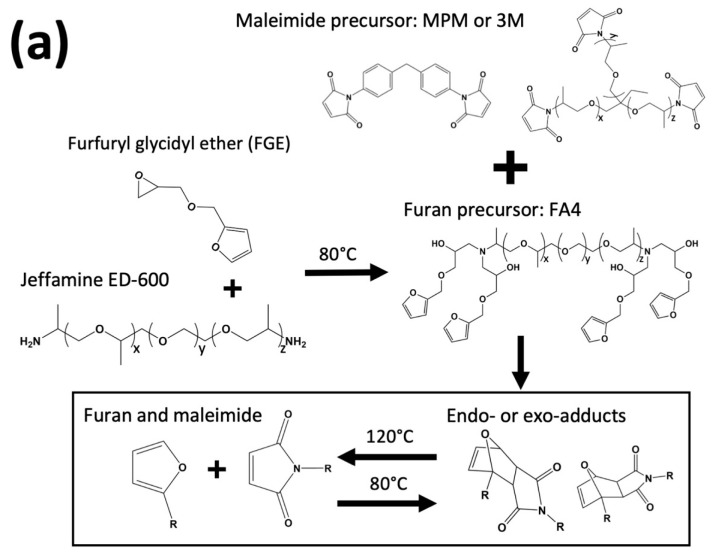

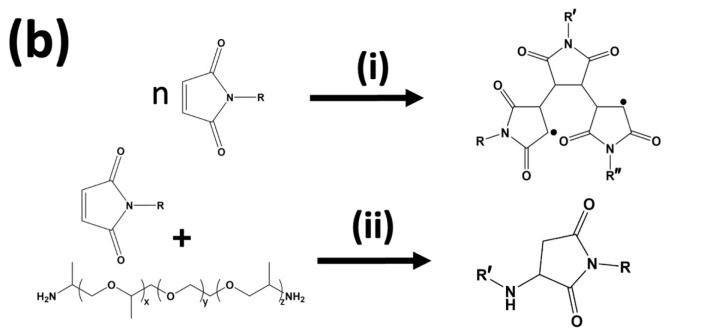
Reaction schemes of (**a**) FA4-MPM and FA4-3M and (**b**) possible side reactions: (**i**) maleimide homopolymerization and (**ii**) Michael’s addition reaction between maleimide and unreacted amine.

In this study, we first confirmed that the irreversible crosslinking in epoxy with DA adducts happening at high temperature is indeed due to the homopolymerization of maleimide. Then, we studied several strategies to reduce the effect of the side reaction. The first strategy was to vary the stoichiometric ratio of furan and maleimide to tune the crosslinking density of the reversible epoxy and to delay the onset of the side reaction. Second, a free radical inhibitor such as hydroquinone was employed to suppress the maleimide homopolymerization, which is known to be a free radical-initiated reaction [17,18,19,20]. Here, we provide a detailed study on the effects of hydroquinone and the side reaction through rheology of the reversible epoxy, which are missing in the literature. The third strategy was to replace a bismaleimide compound with a trismaleimide compound (shown in Scheme 1). In the stoichiometric case, the molar concentration of maleimide groups in the trismaleimide resin is 80% that of the bismaleimide one, reducing the reaction rate of maleimide homopolymerization. Another benefit of having an additional maleimide group on the molecule is that the larger trismaleimide molecules are less mobile. Consequently, the degree of homopolymerization would be further limited by steric hindrance. All these strategies proposed to minimize the maleimide side reaction have significant effects on the rheological and mechanical properties of the reversible epoxy.

## 2. Materials and Methods

Synthesis of DA Polymers. All compounds were purchased from Sigma-Aldrich and used as-is without further purification unless noted. For the furan-functionalization step to produce a four-arm furan prepolymer (FA4), a stoichiometric equivalent of furfuryl glycidyl ether (FGE) was reacted with Jeffamine ED-600 from Huntsman (1.17 g/1.00 g) at 80 °C for 24 h. Completion of the reaction was confirmed by ^1^H NMR. Precursor properties are given in Table S1. Various stoichiometric ratios of maleimide-to-furan (*r* = [M]/[F]) samples were prepared (0.4 to 1.0) using 0.627 g 1,1′-(methylene-4,1-phenylene)bismaleimide (MPM) to 1.00 g of FA4 prepolymer for the *r* = 1 sample. Maleimide and furan concentrations in samples with different ratios are given in Table S2, along with the precise ratios. Additional synthesis details can be found in our previous report [21]. Samples with stoichiometric ratio of 1.0 with hydroquinone were prepared by mixing in chloroform (mix ratio of 1.59:1.00:0.05:10 by weight of FA4:MPM:hydroquinone:chloroform). Hydroquinone and MPM were first dissolved in chloroform followed by the addition of FA4. Solvent was removed using a rotary evaporator followed by vacuum drying at 80 °C for 24 h (~100 torr). The trismaleimide compound (3M) was synthesized by the procedure of Marref et al. [17]. Briefly, 7.29 g mol Jeffamine T-403 (Huntsman, Akron, OH, USA), 4.41 g of maleic anhydride (>99%, TCI, Portland, OR, USA), and 6.2 g anhydrous dimethylformamide were reacted at 115 °C for 10 min under N_2_ purge. Then at 90 °C, 0.6 g triethylamine, 0.03 g of Ni(II) acetate (Acros Organics, NJ, USA), and 6.2 g of acetic anhydride were added and further reacted at 90 °C for 30 min. After cooling down, the mixture was added to 300 mL of chloroform in a separatory funnel, where the organic phase was washed two times with 0.06 M acetic acid, four times with chilled water, and two times with 0.06 M sodium hydroxide. The organic phase was dried with magnesium sulfate, concentrated with a rotary evaporator, and then further dried in vacuum at 50 °C. Typically, yields of 30–50% 3M were achieved.

Fourier Transform Infrared (FTIR) Spectroscopy was performed to confirm the reactions associated with the synthesis, the side reaction, and effect of inhibitors. FTIR spectra were obtained using a Nicolet Is50 FTIR. A DiaMaxATR high thruput heated attachment was used for in-situ temperature experiments. Pressure was applied to the solid samples, and the temperature was set. An omnic macro was written to take samples every ~30 s for the first 15 min to observe the short-time effects. After 20 min, spectra were taken every 10 min to observe long-time effects. Spectra were taken at a resolution of 4 and 16 scans per spectra.

Oscillatory shear rheometry was conducted with a Rheometrics ARES-M instrument. The linear viscoelastic range at different temperatures was determined in the usual manner by strain sweep tests and observing the range of linear torque response. Unless specified otherwise, tests were conducted using 8.0 mm parallel plates at ramps of 2.0 °C/min and 1.0 Hz frequency. Auto-strain was also enabled with a minimum and maximum torque range of 50 to 250–300 μN-m, using up to 100% strain at liquefaction and 50% adjustment of current strain. Since the polymers de-crosslink and soften at elevated temperatures, auto-compression was enabled with 0.0 ± 0.10 N.

Differential scanning calorimetry (DSC) was conducted using a TA Instruments DSC Q2000 instrument with a Refrigerated Cooling Accessory (RCS90) and nitrogen purge gas (50 mL/min). To improve bottom-of-pan contact, samples were flattened with a mortar and pestle. After testing, lids were removed to visually inspect the samples and their contact. Nominal testing parameters were 10 °C/min and 6–12 mg of sample in lidded Tzero aluminum hermetic pans. Temperature and enthalpic calibration were completed with a high-purity indium standard at used ramp rates, and prior to testing samples on a given day, a cooler conditioning was performed along with heat flows zeroed at 0 and 150 °C.

Ultraviolet-visible spectrometry (UV-Vis) scans of the neat MPM and MPM with hydroquinone thin films were performed using a Thermo Scientific Evo260 Ultraviolet Visible Spectrum Spectrometer over the range 300 to 600 nm. A spin coater was used to form MPM thin films using a solution of 10 wt% MPM dissolved in chloroform. The thin films of MPM with hydroquinone were made with a solution of 10 wt% MPM and 1.0 wt% HQ in chloroform. To study the side reaction, the thin films were placed in an oven at 150 °C for different time up to 180 min. Spectra analysis followed the method of Okihara et al. [22].

## 3. Results and Discussion

### 3.1. Effect of Stoichiometric Ratios on the Side Reaction

The DA epoxy was prepared through the reaction steps in Scheme 1a. The FA4 precursors are synthesized by mixing of Jeffamine series ED-600 with furfuryl glycidyl ether (FGE) at 80 °C for 24 h. Completion of the reaction was confirmed by ^1^H NMR (Figure S1). To form the DA thermoset, 1,1′-(methylene-4,1-phenylene)bismaleimide (MPM) or trismaleimide compound (3M) is reacted with the FA4 precursor at 80 °C for 24 h. Scheme 1b shows two possible side reactions associated with the maleimide moiety in the DA epoxy: (i) maleimide homopolymerization and (ii) Michael’s addition reaction of unreacted amine with maleimide.

**Figure 1:**
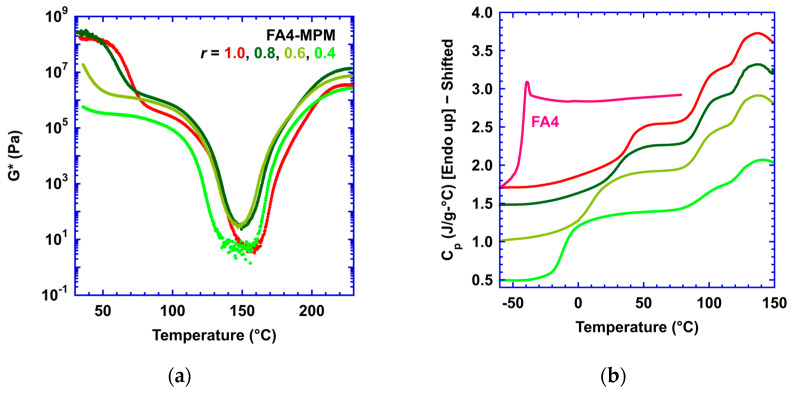
(**a**) Rheological oscillatory shear scans of FA4-MPM samples (*r* = [M]/[F]). Samples were initially cooled from 140 °C to 30 °C at 2 °C/min before heating. (**b**) DSC scans on same samples (second heats, *r* = 0.8, 0.6, and 0.4 shifted down 0.2, 0.66, and 1.04 J/g-°C, respectively).

To identify the side reaction and its effect, the FA4-MPM sample was prepared with different stoichiometric ratios of maleimide-to-furan (*r* = [M]/[F]) with nominal values from 0.4 to 1.0. Maleimide molar concentrations, [M_0_], of the FA4-MPM and the FA4-3M were found assuming volumetric ideal mixing (see Tables S1 and S2). For the different FA4-MPM stoichiometric ratios, [M_0_] was found to be range between 1.25 mol/L (for *r* = 0.4) and 2.51 mol/L (for *r* = 1.0). Figure 1a shows the rheology data and Figure 1b shows the DSC data for thermosets in which the FA4 and MPM ratio was varied.

As temperature increased, softening of the polymer occurred first due to glass transition and followed by flow behavior due to the depolymerization by the rDA reactions. As the ratio of maleimide to furan decreased, shear moduli dropped, and the glass transition temperature (*T*_g_) decreased because of reduced crosslinking density. Interestingly, heating past 150 °C, a rapid increase in the shear moduli occurred. At these elevated temperatures (>150 °C), the thermomechanical behavior changed significantly, restricting further flowability of the polymers. If the polymer was cooled from high temperature (Figure 1a), the modulus remained glassy during the temperature ramp indicating that permanent crosslinking occurred. The NMR data of furan precursor confirmed there was no unreacted amine in the system (Figure S1), eliminating the possibility of a Michael addition reaction between maleimide and unreacted amine. Therefore, the irreversible crosslinking observed in this system may be attributed to the formation of succinimide chains from self-polymerization of maleimide [23]. This is also later confirmed from FTIR data. A prior DSC study [9] on neat MPM showed homopolymerization beginning just above the melting point of 162 °C [24] and peaking at ~230 °C in a heating ramp of 10 °C/min. Isothermal rheology on MPM showed [9] a significant viscosity increase after 8 min at 183 °C. In brief, prior DSC and rheology studies show that neat MPM homopolymerization is clearly observed for temperatures above its melting point (162 °C). In the DA epoxy, we find that maleimide homopolymerization can occur at lower temperatures because of the lack of crystallinity.

Figure 1a clearly shows the effect of varying the maleimide-to-furan ratio, *r*, on the rheology of reversible epoxy and onset of irreversible crosslinking by the side reaction. In the rheology, the *T*_g_ expectedly is higher for the higher stoichiometric samples, but the lower ratios are stiffer in the rubbery region (*r* = 1.0 vs. *r* = 0.6 and 0.8). The origins of this behavior could be attributed to the dynamic interplay between mobility and the extent of side reaction at higher temperatures. Lower ratios have higher mobility (lower T_de-gel_), and because these samples were initially cooled from 140 °C (Figure S2), the extent of side reaction could be increased for the lower ratios. In the case of the lower maleimide to furan ratio (*r* = 0.4) sample, we see a delayed onset of the irreversible crosslinking. At this low ratio of maleimide to furan, it is reasonable to expect that the concentration of MPM is sufficiently low to diminish the effects of the side reaction of maleimide. For the same reason, we do not expect the side reaction in elastomeric materials prepared with little (maleimide) crosslinker to have a pronounced effect on their recyclability. For example, for rubber materials, which are generally prepared with minimal crosslinker (<5%) to strengthen but retain flexibility, we suspect the side reaction would be sluggish even at elevated temperatures (120 °C or more) because of the reduced mobility and low maleimide concentration. Rubbers can remain stiff at elevated temperatures because of their high molecular weights (50,000+ g/mol) and resulting chain entanglements. For instance, in one study using DA rubbers, the authors used between 0 and 8.1 wt% bismaleimide precursor as crosslinker for the furan-grafted rubbers, whose shear moduli remained around 0.01 MPa across 120–180 °C implying that no significant maleimide self-reaction occurred [25].

Thermal analysis is sensitive and can also inform on the structure of DA adducts. Two isomers of the DA adduct exist (“endo-” and “exo-”) of differing stability, leading to separate activation energies and temperatures at which de-bonding occurs [7,8,13,16,17,20,26,27]. The two high temperature endothermic features seen in the DSC thermograms of Figure 1b correspond to the rDA reactions of the endo- and exo-stereoisomers, which parallel the rheology of Figure 1a. As the temperature is ramped from low to high, the FA4-MPM samples pass the glass transition which increases in temperature with the stoichiometric ratio *r* (see Table 1 below). Subsequently, for all FA4-MPM samples regardless of *r* value, a shoulder in the thermograms appears at ~100 °C and a peak occurs at ~140 °C due to the rDA reactions of each stereoisomer. This temperature range (100 °C to 140 °C) corresponds to the rapid drop in modulus in Figure 1a as the network depolymerizes. In addition, the magnitude of the endo- and exo-peak areas correlates with the stoichiometric ratio *r*, since the larger the *r*, the greater the concentration of DA bonds for the rDA reaction to de-bond.

**Figure 2:**
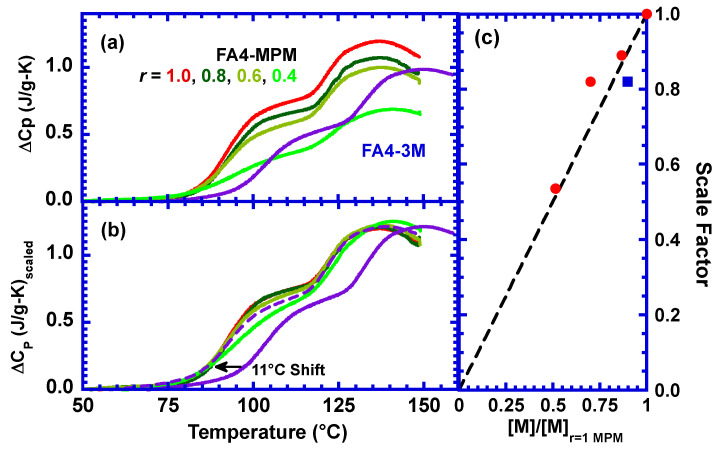
Heat capacity difference from value just above *T*_g_. This is shown both (**a**) for the un-scaled thermograms in ascending order of r for FA4-MPM (0.4, 0.6, 0.8, 1.0) and for FA4-3M with rDA response beginning 11 °C higher, and (**b**) for the scaled thermograms where division by a scale factor results in overlaying FA4-MPM (1.0) with colors corresponding to (**a**), and FA4-3M shifted 11 °C to the left, as shown by the dashed purple line. (**c**) Scale factor vs. ratio of maleimide concentration for both FA4-MPM (red dots) and FA4-3M (blue square). Line has a slope of 1. Precise values of r used to calculate [M]: (0.384), (0.573), (0.777), (0.980).

This can be quantified by shifting and scaling the different thermograms (from Figure 1a to Figure 2a) so that the rDA response regions overlay over 50–150 °C. This scale factor is the relative heat capacity response due to the rDA reaction, and this ratio should be proportional to the concentration of DA groups which, in turn, is expected to be proportional to the molar concentration of maleimide groups. A plot of the scale factor vs. the ratio of maleimide concentration to that of the FA4-MPM (*r* = 1.0) is shown in Figure 2c. The resulting linear relationship supports the abovementioned interpretation of the thermograms (i.e., rDA reactions occur predominantly at these temperatures, and not some other thermal event). Additionally, to compare the effect of trismaleimide (3M) and bismaleimide (MPM) compounds, the FA4-3M thermogram was shifted by 11 °C and scaled to overlay the FA4-MPM(*r*) case, which shows excellent agreement. This 11 °C delay in the onset of the rDA reaction is likely the result of differing flexibility of the MPM and 3M reactants. A more detailed analysis of this behavior would involve the variation of the rDA/fDA equilibrium coefficient [2]. For the further studies, all DA polymers were prepared with the equal molar stoichiometric ratio, i.e., *r* = 1.0.

Table 1 shows a summary of softening points from the abovementioned DSC and rheology data. Figure S3 shows DSC and rheology data run in triplicate; for measurements from the same instrumentation and same technique, *T_g_* values do not vary more than 2–3 °C. Variation as large as 10 °C or more of the *T_g_* among DSC and Rheometry data as shown in Table 1 is not unexpected. The glass transition is a “dynamic” transition and sensitive to measurement techniques and instrumentation. Hence, meaningful comparison of the *T_g_*’s of two polymers requires the same type of instrument (e.g., DSC) and experimental protocol. In a previous report in which we used a similar Jeffamine base material, we found *T_g_* to vary by up to 20 °C [28]. Listed in the table also is a “flow” temperature which corresponds to a complex viscosity of 10,000 Pa-s from the abovementioned rheology data. There have been a number of papers using the complex viscosity from rheometry to guide experiments with inkjet 3D printing of raw epoxy or DA resins [29,30,31]. Our laboratory standard of 10,000 Pa-s as the flow point is based on the maximum viscosity for the printing of similar polymeric resins.

**Figure 3:**
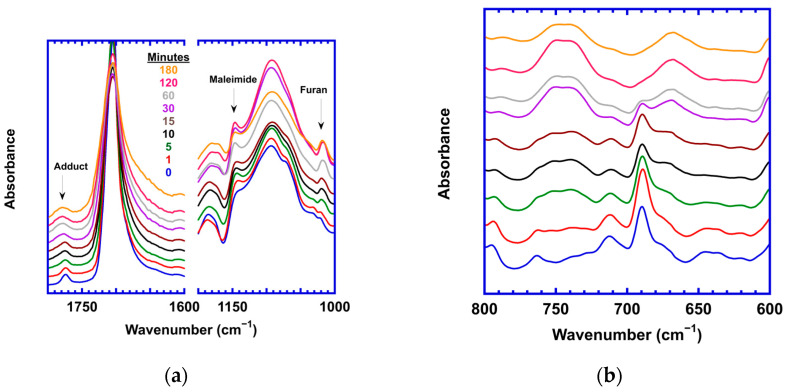
FTIR spectra for FA4-MPM sample treated for different time at 150 °C (**a**) from 1800 cm^−1^ to 1000 cm^−1^ and (**b**) from 800 cm^−1^ to 600 cm^−1^ representing changes in maleimide moieties exclusively, demonstrating the evolution of the side reaction over 3 h.

To understand the effect of the side reaction, we pursued detailed FTIR studies. Figure 3 shows FTIR spectra of FA4-MPM sample treated for different time at 150 °C, where the zero time was taken when the temperature stabilized at 150 °C. The absorption peak at 1775 cm^−1^ is indicative of DA adducts [32]. The furan and maleimide concentrations were monitored by observing the change in the peak at 1010 cm^−1^ in Figure 3a and the peak at 690 cm^−1^ in Figure 3b, respectively [33]. At 150 °C, the rDA reactions occurred rapidly to depolymerize the DA network and free up a portion of the furan and maleimide from the adducts. Interestingly, over time at 150 °C, the DA adduct peak continued to decrease and the furan peak increased, suggestive of ongoing reactions. Ultimately, the magnitudes of these peak areas plateaued probably due to vitrification and the resulting frozen fDA/rDA equilibrium. In addition, the maleimide peak at 690 cm^−1^ increased initially due to the free maleimide resulting from rDA reaction, but then continuously decreased. Continuous and rapid consumption of maleimide moieties over time implies the evolution of a side reaction at 150 °C.

**Figure 4:**
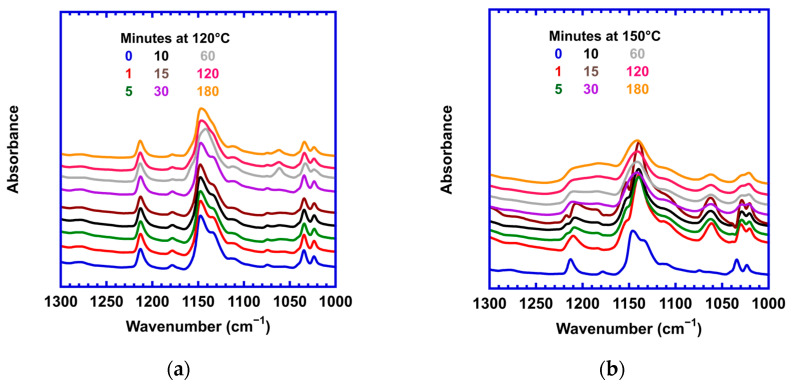
FTIR spectra for MPM powder after treatment under vacuum for different period of time at (**a**) 120 °C and (**b**) 150 °C.

To further confirm the nature of the side reaction, pure MPM powder was analyzed with FTIR after treatment at two different temperatures 120 °C and 150 °C at different times (as shown in Figure 4). The maleimide peak near 1140 cm^−1^ from Figure 3 can also be attributed to the C-N-C stretch, which shifts to 1180 cm^−1^ in forming succinimide moieties generated due to free radical-initiated homopolymerization of maleimide [34]. The spectra of the MPM sample exposed to 120 °C in Figure 4a showed no visible evolution of a succinimide peak. However, MPM samples treated at 150 °C showed gradual evolution of a broad peak at 1180 cm^−1^ indicating the formation of succinimide. Other spectral changes are observed, such as the merging of doublet peaks at 1145 cm^−1^; however, additional changes could also be attributed to phase change as the sample becomes glassier during homopolymerization.

### 3.2. Effect of a Free Radical Inhibitor on Side Reaction

**Figure 5:**
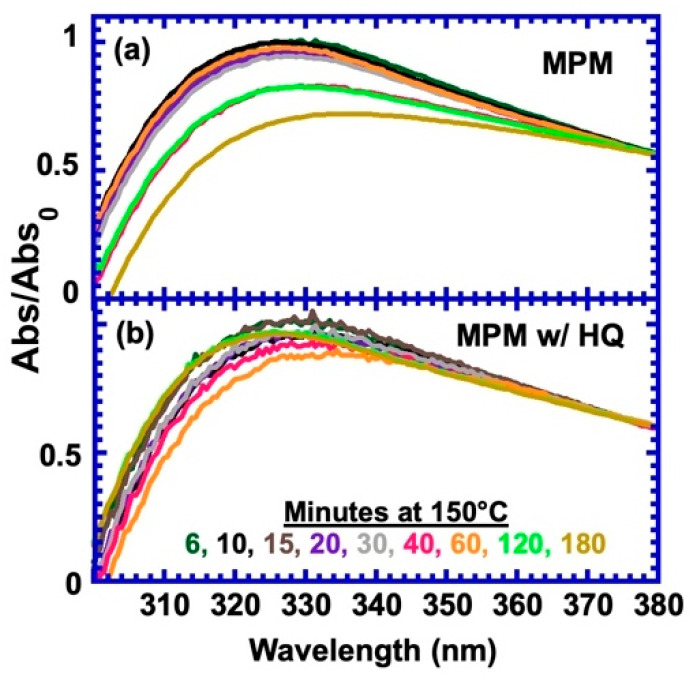
UV-Vis spectra were taken of MPM thin films (**a**) without and (**b**) with hydroquinone (HQ) at 150 °C over time to track progression of MPM homopolymerization. Spectra are divided by a constant to make peak height at early time equal to one, and a constant is added so spectra are equal at 380 nm following the method of Okihara et al. [22]. Spectra were taken at 6, 10, 15, 20, 30, 40, 60, 120, and 180 min.

Maleimide homopolymerization is a radical-initiated polymerization and can be inhibited by using a radical scavenger such as hydroquinone [35]. Quinones are known for readily accepting or donating free radicals to form a more stable radical species [36]. To confirm hydroquinone’s inhibiting ability, we first studied the effect of hydroquinone on neat MPM reactions. We used UV-Vis spectroscopy to track the progression of MPM homopolymerization with and without hydroquinone over time at 150 °C (Figure 5). The UV-Vis absorbance peak of maleimide groups without hydroquinone is in the range 290 to 310 nm depending on molecular details [22,37,38]. We found the maleimide peak for MPM to be slightly higher at 325 nm and to gradually decrease as maleimide groups were consumed by homopolymerization until the sample became glassy and the reactions became diffusion-limited. The addition of hydroquinone slowed down the homopolymerization as shown in Figure 5b.

We further studied the effect of hydroquinone on maleimide homopolymerization in the reversible FA4-MPM polymer with DA adducts. Absorbance peak areas assigned to the maleimide, furan, and DA cycloadduct in FTIR spectra were tracked over time when the sample was subjected to 150 °C. Peak areas were then normalized to values between 0 and 1 for maleimide, furan, and adduct peaks using

(1) $$A_{n}=\frac{A-A_{min}}{A_{max}-A_{min}}$$

*A_n_* is the normalized peak area absorbance, *A* is the peak area absorbance at a certain time, *A_min_* is the minimum peak area absorbance observed during the studied time frame, and *A_max_* is the maximum peak area absorbance observed.

**Figure 6:**
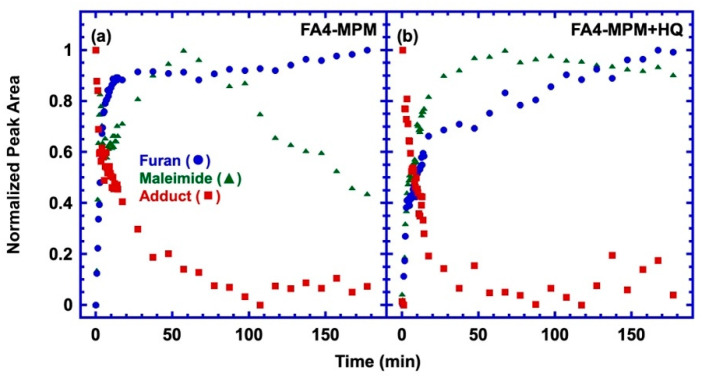
FTIR of FA4-MPM (**a**) without and (**b**) with hydroquinone (HQ) at 150 °C showing evolution of normalized peak areas.

Figure 6a,b show that the presence of hydroquinone does not change the rate of adduct consumption and furan production. Within the first 20 to 30 min, the rapid decrease of cycloadducts, or rapid increase of free furans and maleimides, is because of the rDA reaction being predominant over fDA at these temperatures, but by tracking the long-time behavior of the maleimides, hydroquinone’s inhibiting ability is confirmed. The concentration of maleimide did not visibly change after ~30 min in samples containing hydroquinone, whereas samples without hydroquinone had a decreasing concentration. As expected, these results demonstrate the ability of hydroquinone to reduce the rate of maleimide homopolymerization in reversible Furan–maleimide systems.

**Figure 7:**
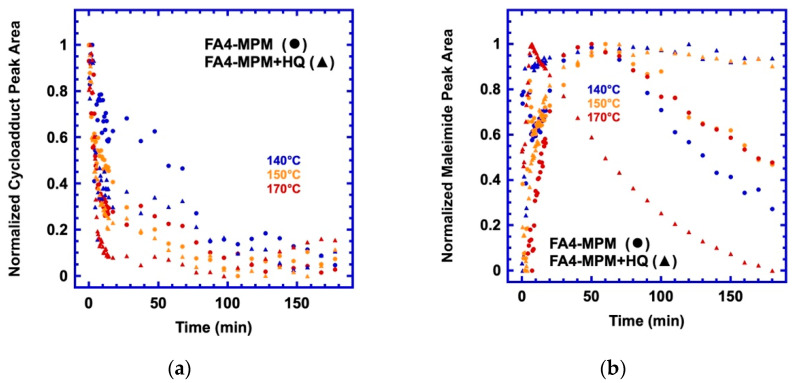
FA4-MPM with and without hydroquinone at different temperatures over time for (**a**) normalized DA adduct peak area and (**b**) normalized maleimide peak areas.

We further studied reactions of an epoxy polymer with DA adducts with and without hydroquinone at different temperatures. Figure 7 shows the normalized peak area of DA cycloadduct and MPM of the FA4-MPM samples with and without hydroquinone treated at 140, 150, and 170 °C. We did not see any specific effect of hydroquinone on cycloadduct conversion (Figure 7a). Interestingly, the addition of hydroquinone slowed down the consumption of maleimide due to the side reaction at both 140 °C and 150 °C as expected; however, it had the reverse effect on maleimide homopolymerization at 170 °C (Figure 7b). Although beyond the scope of this paper, the anomalies in trend observed at 170 °C could be due to other reactions happening between the precursors of DA adducts and hydroquinone. However, a potential explanation is that the radicals on hydroquinone become active again because quinones with their resonance structures only stabilize radicals, not eliminate them.

### 3.3. Effect of a Different Maleimide Precursor on the Side Reaction

Another strategy used to minimize the side reaction was to employ a new maleimide precursor. We replaced the bismaleimide compound (MPM) with a trismaleimide compound (3M); the chemical structures are shown in Scheme 1a. MPM has two maleimide functional groups per molecule of MW 358 g/mol while 3M has three maleimide functional groups per molecule of MW 726 g/mol which reduces the maleimide concentration [M] by about 20% (Table S2). The reduced [M] is expected to reduce the rate of homopolymerization. In addition, the greater size of 3M molecules is anticipated to reduce the molecular mobility, which would also adversely affect the reaction rate.

**Figure 8:**
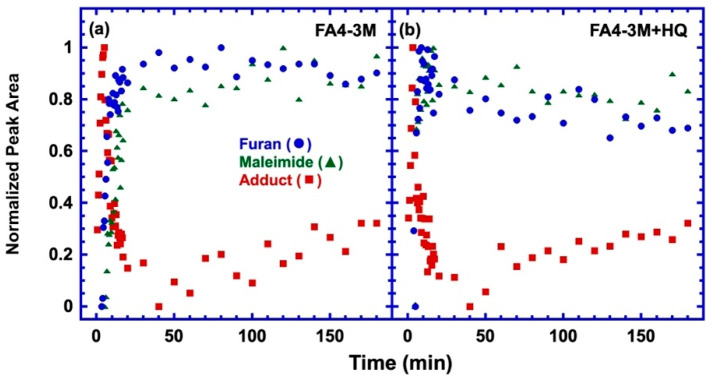
FTIR of FA4-3M (**a**) without and (**b**) with hydroquinone (HQ) at 150 °C showing evolution of normalized peak areas.

As designed, the use of 3M significantly adversely affects the onset of side reaction at 150 °C (Figure 8a). In contrast to FA4-MPM in Figure 7b, the free maleimide concentration increases along with the furan concentration at the beginning due to rDA reactions, but there is no noticeable decrease even after three hours. In samples without (Figure 8a) and with hydroquinone (Figure 8b), the adduct concentration decreased during heating due to predominance of rDA over fDA reactions at 150 °C. For the time scale studied, comparison of the maleimide concentration between samples without and with the addition of hydroquinone presented no significant effect of hydroquinone on the side reaction.

**Figure 9:**
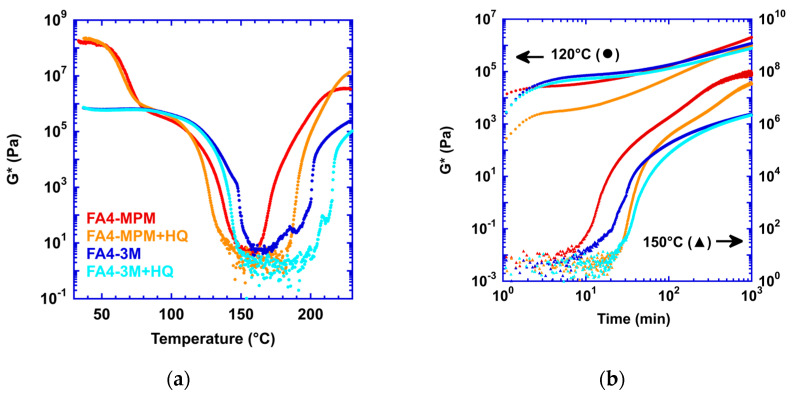
Complex moduli (G*) of FA4-MPM and FA4-3M samples without and with hydroquinone (HQ) from (**a**) high-temperature scan up to 230 °C and (**b**) isotherm tests at 120 °C and 150 °C.

Hydroquinone is a known free radical inhibitor and is commonly included into these polymeric Furan–maleimide chemistries to prevent maleimide homopolymerization; however, its effect on rheology to the best of our knowledge has not been reported [17,18,19,20,39]. In Figure 9, the samples with hydroquinone demonstrate comparatively less stiffness overall compared with the samples without. While this could be in part due to hydroquinone and chloroform plasticization in the FA4-MPM and FA4-3M networks, with support of Figure 9b, a better explanation is that hydroquinone is serving its intended purpose of stabilizing free radicals of the maleimide and delaying the onset of irreversible crosslinking.

Briefly, the tests conducted at 150 °C were taken straight from room temperature, but the 120 °C tests were first jumped to 140 °C to de-gel the samples by rDA, adhere the samples to the plates, and then measure the properties as the temperature stabilized at 120 °C. Ensuring that samples adhere and are flush between the plates takes about 1–2 min. At 120 °C, besides just being at a lower temperature and slower free radical generation, the crosslinking due to equilibrium rDA/fDA reactions likely reduces the mobility, compared with 150 °C where many free radicals can be generated and move around freely in the molten state. Yet, at 120 °C, the power law slopes change noticeably for all the samples past 10 to 100 min. Since the activation energy barrier for rDA reactions must be higher than that of the fDA reactions, any increases in temperature from there will accelerate the frequency of rDA more than that of fDA, reducing DA crosslinks (i.e., equilibrium shifts in the rDA direction) [40]. Therefore, this change in power law slopes could not be attributed to a relative increase of the fDA reaction at these temperatures.

The FA4-3M is softer compared with FA4-MPM even after the homopolymerization since the maleimide units exist on a flexible polymer backbone. It can be argued that the observed material behavior of FA4-MPM and FA4-3M is the result of a blend network, with both reversible and permanent bonds contributing to the network stiffness. By examining the structures of MPM and 3M, one could also reason that MPM will serve as a stiffer backbone due to its phenyl rings. This would help explain why so little MPM must react to irreversibly stiffen the network (compare 10 min in Figure 5 and Figure 9b). Our rheology data confirm that 3M compared with MPM reduces the extent of network stiffening by the side reaction. This could be due to several reasons: trismaleimide (1) is heavier so diffusion is slower; (2) has fewer maleimide groups per volume; (3) has different reactivity; and (4) has less adduct bond stiffness when a succinimide unit or chain forms.

**Figure 10:**
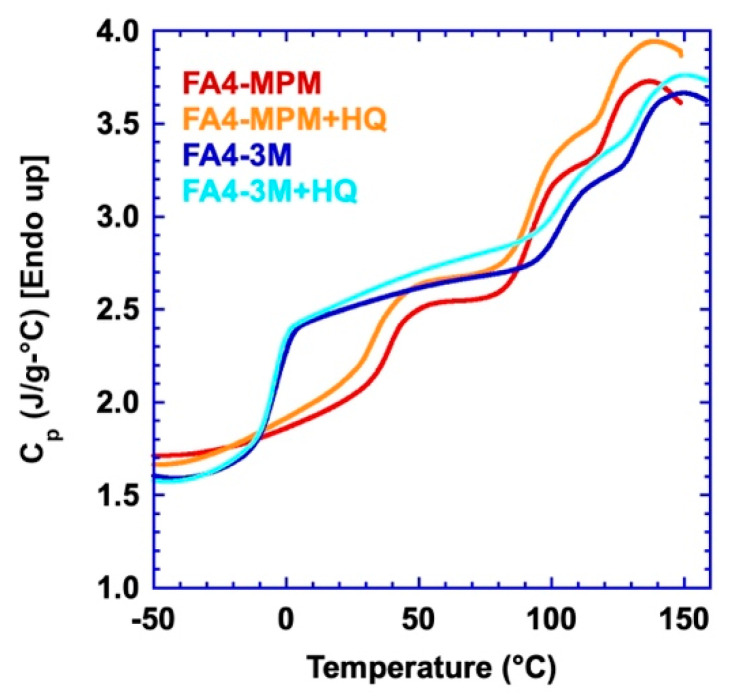
DSC scans on FA4-MPM and FA4-3M without and with hydroquinone (HQ) (2nd heats).

In Figure 10, the temperature ranges differ for thermal events among the DA polymers and the samples with hydroquinone on the second heat. Expectedly, since the FA4-3M samples have maleimides functionalized onto a longer polymer backbone (Jeffamine T403), the glass transition temperature is lower compared to that of the FA4-MPM samples. Second, the rDA heat flows past 90 °C occur at higher temperatures for the FA4-3M compared to the FA4-MPM. This suggests the FA4-3M DA adducts have a higher activation energy barrier for rDA reactions, which could be because of a few reasons. Specifically, 3M adducts are more flexible, and MPM has its maleimide groups neighboring phenyl rings that can pull additional electron density; thus, the thermodynamic stability between DA adducts formed by MPM and 3M should vary [3].

## 4. Conclusions

Diels–Alder polymers formed via Furan–maleimide chemistry are among the most common dissociative covalent adaptable networks studied for their thermoreversibility; general reasons include their ease of synthesis and recyclability without byproducts (e.g., water), expensive catalysts, or additional stimuli (e.g., pH) [41]. This chemistry shows significant promise for producing a range of useful sustainable thermoplastics and thermosets because of reports on their adhesive, self-healing, and 3D-printing qualities [3,42].

An important question probed through this work by overall reaction kinetics and rheological analyses was how long such DA materials can remain recyclable at elevated temperatures. Retro-DA occurs above 110 °C to return the polymer network into a (re-)processable melt state. However, at that high temperature, there is a possibility of a side reaction to form irreversible crosslinking through the maleimide homopolymerization. Applications of thermoset polymers typically require their robust mechanical property and thermal stability at operating conditions. To fulfill these requirements on top of the reversibility of thermosets, reversible (or recycle) processes at high temperature (e.g., >160 °C) with reduced side reaction is very important. The small-molecule bismaleimide compound MPM studied here, which is among the most commonly used maleimides [2,19,23,26,35,39,43], suffered from rapid network stiffening and lack of recyclability due to side reactions. The onset of this network stiffening was observed at 160 °C in a temperature sweep and 10 min as a function of duration at 150 °C.

A number of mitigation strategies against maleimide homopolymerization were explored. Reducing the side reaction by changing the feed ratio of reactants is one possibility. However, this is not a linear relationship probably because of the network mobility increase associated with smaller ratios and less crosslink density [20,39]. We also provided a detailed study on how adding a radical-reaction inhibitor, hydroquinone, could regard the side reaction. Further, in addition to having greater functionality with a larger molecular weight than bismaleimide, the new maleimide precursor has slower diffusion and lower concentration in the system, both of which would slow the onset of the side reaction and reduce its consequent network stiffening. Complete prevention of the maleimide homopolymerization may not be feasible especially in systems in which the (re-)processing temperatures exceed 150 °C, such as in rubber systems utilizing the DA chemistry [25,44]. Nevertheless, these strategies are all potential ways to minimize the impact by the maleimide side reaction and provide insight into how to extend the service life over repeating process cycles of such materials.

## Figures and Tables

**Table 1:** Summary of characteristic points in thermomechanical behavior of FA4-MPM samples with different stoichiometric ratios.

Stoichiometric Ratio *r* = [M]/[F]	Calorimetric *T_g_*	Rheological *T_g_*	Flow Temp (Visco. = 10^4^ Pa-s)	Temp. of Min G*	[M] (mol/L)
0.4	−13	NA	97	148	1.26
0.6	8	38	116	147	1.74
0.8	31	61	119	150	2.15
1.0	38	70	111	157	2.51

## Data Availability

The data presented in this study are available on request from the corresponding authors.

## Supplementary Materials

The following supporting information can be downloaded at: https://www.mdpi.com/article/10.3390/polym15051106/s1. Table S1 provides property table with molecular weight, specific volume, and molar volume of different precursors. Table S2 includes molarity of furan and maleimide moieties in the DA polymers. Figure S1 provides ^1^H-NMR spectra and assignments of FGE, Jeffamine ED-600, and FA4. Figure S2 shows complex viscoelastic modulus and Tan(δ) for samples corresponding to Figure 1a and Figure 9a. Figure S3 is DSC and rheology data in triplicate demonstrating reproducibility.
