# Supplementary material for: Understanding the Effect of Side Reactions on the Recyclability of Furan–Maleimide Resins Based on Thermoreversible Diels–Alder Network
Source: Polymers (Basel). 2023 Feb 23;15(5):1106. doi: 10.3390/polym15051106 (PMC10007558; doi:10.3390/polym15051106)
Supplement: Supplementary file 1 [file polymers-15-01106-s001.zip › polymers-2199989-supplementary.pdf]

polymers

MDPI

*Supplementary Information*

# Understanding the Effect of Side Reactions on the Recyclability of Furan-Maleimide Resins Based on Thermoreversible Diels-Alder Network

**Brandon T. McReynolds [1,†], Kavon D. Mojtabai [2,†], Nicole Penners [2], Gaeun Kim [2], Samantha Lindholm [2], Young-min Lee [2], John D. McCoy, and Sanchari Chowdhury [2,*]**

[1] Department of Chemical Engineering
[2] Department of Materials and Metallurgical Engineering
New Mexico Institute of Mining and Technology, Socorro, NM
* Correspondence: sanchar.chowdhury@nmt.edu, youngmin.lee@nmt.edu, and john.mccoy@nmt.edu
† These authors contributed equally to this work.

**Citation:** To be added by editorial staff during production.

Academic Editor: Firstname Last-name

Received: date
Revised: date
Accepted: date
Published: date

**Copyright:** © 2023 by the authors. Submitted for possible open access publication under the terms and conditions of the Creative Commons Attribution (CC BY) license (https://creativecommons.org/licenses/by/4.0/).

**Table S1.** Property table with molecular weight, specific volume, and molar volume

| Precursor | Molar Mass (g/mol) | Specific Volume (mL/g) | Molar Volume (mL/mol) |
|---|---|---|---|
| ED-600 | 528 | 0.966 | 510 |
| T-403 | 486 | 1.02 | 496 |
| FGE | 154 | 0.891 | 137 |
| FA4 | 1145 | 0.926 | 1060 |
| MPM | 358 | 0.749 | 268 |
| 3M | 726 | 0.890 | 646 |

**Table S2.** Molarity of furan and maleimide moieties in the DA polymers

| DA Polymer (r = nominal) | [M] (mol/L) | [F] (mol/L) | Precise r = [M]/[F] | [M] (mol/L) | [F] (mol/L) |
|---|---|---|---|---|---|
| FA4-MPM(0.4) | 1.26 | 3.14 | 0.384 | 1.21 | 3.16 |
| FA4-MPM(0.6) | 1.74 | 2.90 | 0.573 | 1.68 | 2.93 |
| FA4-MPM(0.8) | 2.15 | 2.69 | 0.777 | 2.10 | 2.71 |
| FA4-MPM(1.0) | 2.51 | 2.51 | 0.968 | 2.45 | 2.53 |
| FA4-3M(1.0) | 2.08 | 2.08 | ~1.0 | | |

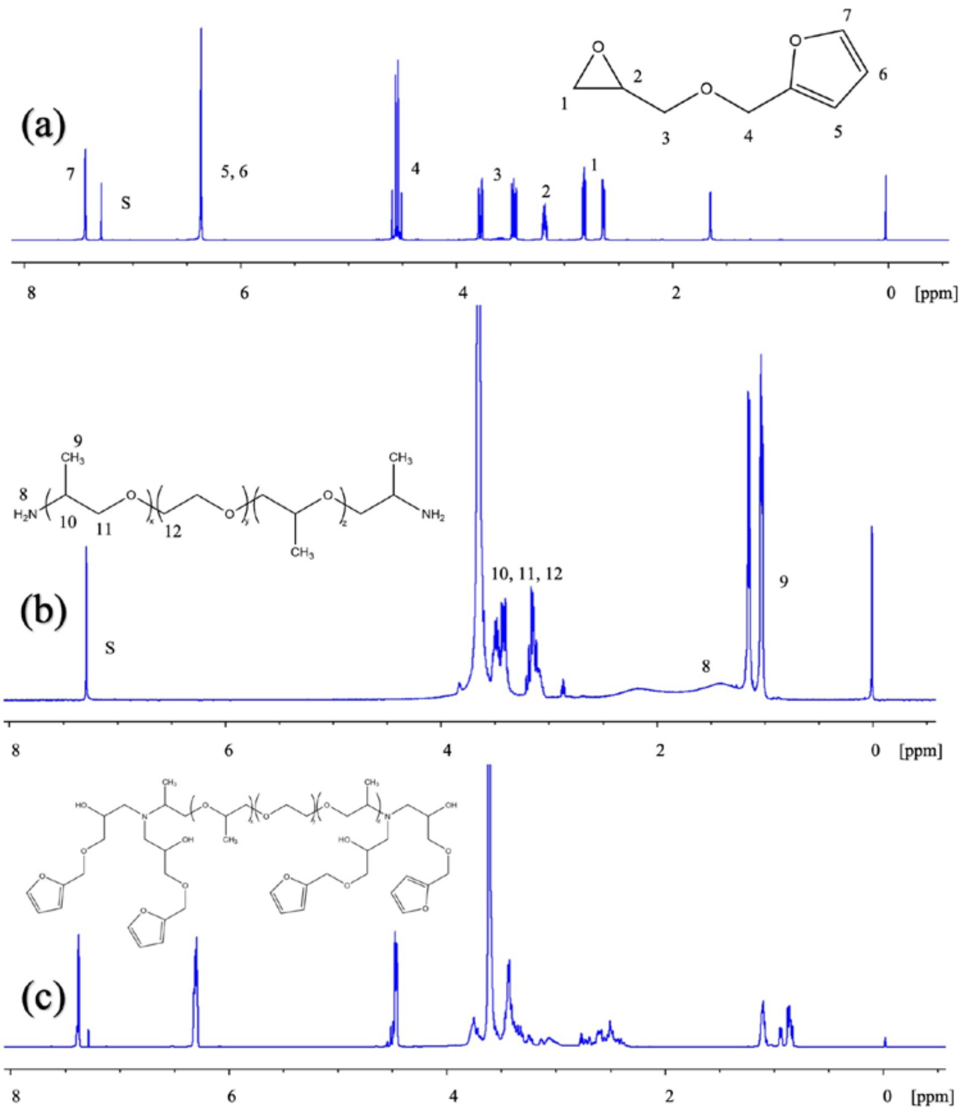


**Figure S1.** $^{1}$H-NMR spectra and assignments of **(a)** FGE, **(b)** Jeffamine ED-600 and **(c)** FA4.

NMR scans were performed using a Bruker Avance III 400 in deuterated chloroform solution. **Figure S1** represents the $^{1}H$-NMR spectra of FA4 and its precursors. The broad signal present from 1.2 ppm to 1.75 ppm in Jeffamine ED-600 is attributed to the amine hydrogens. It is crucial that the epoxy-amine reaction is complete as the unreacted amine can undergo a Michael addition reaction with maleimide to form an irreversible network. The amine chemical peak shift cannot be observed in FA4, signifying that the epoxy-amine reaction is complete.

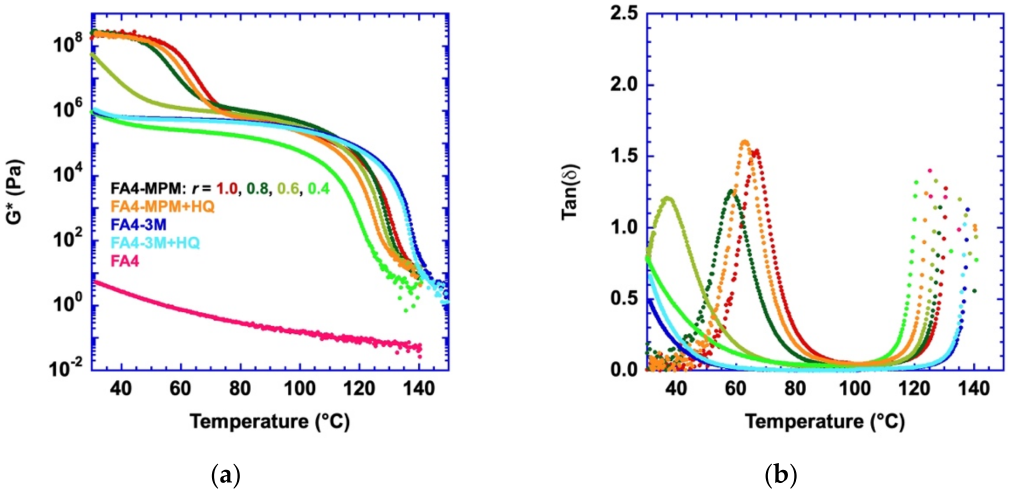


**Figure S2.** Samples corresponding to **Figure 1(a)** and **Figure 9(a)** that were initially cooled from 140 – 150°C plotting **(a)** Complex viscoelastic modulus and **(b)** Tan($\delta$).

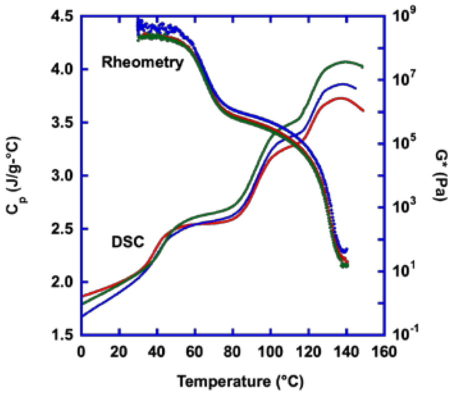


**Figure S3.** FA4-MPM samples run in triplicate to confirm reproducibility of characteristic features from DSC (10°C/min) and rheometry (2°C/min)
